# Supplementary material for: Ursolic Acid Inhibits Collective Cell Migration and Promotes JNK-Dependent Lysosomal Associated Cell Death in Glioblastoma Multiforme Cells
Source: Pharmaceuticals (Basel). 2021 Jan 26;14(2):91. doi: 10.3390/ph14020091 (PMC7911358; doi:10.3390/ph14020091)
Supplement: Supplementary file 1 [file pharmaceuticals-14-00091-s001.zip › Conway et al_Supplemental data.docx]

Ursolic acid inhibits collective cell migration and promotes JNK-dependent lysosomal associated cell death in glioblastoma multiforme cells

Gillian E. Conway^1, 2,3,^ Deimante Zizyte^1^, Julie Rose Mae Mondala ^1^, Zhonglei He^1,2^, Lorna Lynam^1^, Mathilde Lecourt^1^, Carlos Barcia^4^, Orla Howe^2, 5^, James F. Curtin^1, 2^

**Supplemental Figure 1**

Cytotoxic evaluation of UA in cancer cell lines.

| Cell Line | IC_50_ (µM) |
| --- | --- |
| A549 | 12.86 |
| A431 | 17.68 |

**Figure S1:** Dose reponse curves for (A) A549 and (B) A431 cells exposed to increasing concentrations of UA for 48 hours. Cell viability was assessed using Alamar blue cell viability assay for 48 hours (n=3). IC_50_ values were calculated for both A549 and A431 cells using Prism, GraphPad.
